# A Fresh Look at the Unconscious Thought Effect: Using Mind-Wandering Measures to Investigate Thought Processes in Decision Problems With High Information Load

**DOI:** 10.3389/fpsyg.2021.545928

**Published:** 2021-06-24

**Authors:** Lena Steindorf, Jan Rummel, C. Dennis Boywitt

**Affiliations:** ^1^Department of Psychology, Heidelberg University, Heidelberg, Germany; ^2^Independent Researcher, Berlin, Germany

**Keywords:** mind wandering, task-unrelated thought, Unconscious Thought Effect, consciousness, unconscious thought advantage

## Abstract

Unconscious Thought Theory (Dijksterhuis, 2004) states that thinking about a complex problem unconsciously can result in better solutions than conscious deliberation. We take a fresh look at the cognitive processes underlying “unconscious” thought by analyzing data of 822 participants who worked on a complex apartment-evaluation task in three experiments. This task’s information-presentation and evaluation parts were separated by different kinds of filler-interval activities, which corresponded to standard conscious-thought and unconscious-thought manipulations. Employing experience-sampling methods, we obtained thought reports during and after filler-interval engagement. Evidence concerning the existence of the Unconscious Thought Effect was mixed, with such an effect being present in the first two experiments only. In these experiments, we further found less problem deliberation to be associated with better performance on the apartment task. Interestingly, this benefit disappeared when we probed participants’ thoughts during the filler interval. We suggested that explicit thought awareness diminishes the Unconscious Thought Effect.

## Introduction

Life is full of situations requiring decisions. Some of them are rather simple, others are more complex. What should I make for dinner? Which college should I go to after high school? Should I buy this washing machine or another one? From a layman’s perspective, it sounds reasonable that in such situations serious, conscious deliberation should help us make good and satisfying choices. At the same time, other people might argue that – faced with a difficult decision – one should rather sleep on it, or at least stop thinking about it for a while, to get a fresh look at the situation or let our intuition guide us. Especially for complex decisions, Unconscious Thought Theory (UTT, Dijksterhuis et al., 2006; Dijksterhuis and Nordgren, 2006) recommends using the latter strategy. Unconscious thought is supposed to lead to better and more satisfying decisions when choosing, for example, between four apartments, which are characterized by a multitude of attributes. In the present work, we took a closer look at thought processes during conscious- and supposedly unconscious-thought intervals by applying methods used in current mind-wandering research within a standard UTT paradigm. In three experiments, retrospective thought protocols as well as thought reports collected online via thought probes offered insights into the cognitive processes leading to decisions within a complex apartment-evaluation task.

Unconscious thought, which is thought or processing in the absence of conscious attention being directed toward a pending problem, was proposed as a separate form of thought distinct from and, in specific situations, superior to conscious thought (Dijksterhuis and Nordgren, 2006). In a typical UTT experiment, participants are introduced to several objects (e.g., apartments) which are characterized by a specific number of positive and negative attributes per object (e.g., “Apartment 1 has a balcony.”). The objectively best object possesses a relatively high number of positive attributes, the objectively worst object a relatively high number of negative attributes. Before evaluating the objects, participants face a distraction-task period or a period of conscious thought about the presented objects. Evidence for the *Unconscious Thought Effect* (UTE) comes in form of better decisions after distraction periods as compared to conscious thought periods. This effect occurs particularly in complex decision situations, that is, when objects are described by a high total number of attributes, for instance. According to the UTT, the power of the unconscious stems from its high information-processing capacity. The unconscious system is supposed to allow for large amounts of information to be integrated, whereas the conscious system suffers from a low information-processing capacity (e.g., Miller, 1956; Nørretranders, 1998). The latter refers to task-related cognitive processes that one is consciously aware of during task completion and has the advantage over the unconscious system of being rule-based and very precise (Dijksterhuis, 2004). Consequently, when faced with simple decisions, the conscious system’s capacity is not exceeded and our choice benefits from rule-based cognition. We are able to consciously process all available information, which should result in the best possible decision. When faced with complex decision problems, however, its low information-processing capacity renders the conscious system less efficient because not all available information can be processed simultaneously. In these situations, decisions should benefit from the ability of the unconscious system to integrate a high number of decision-relevant attributes. Indeed, unconscious-thought advantages are most prevalently found for complex decision problems (e.g., Dijksterhuis et al., 2006; Strick et al., 2011).

Some assumptions of the UTT have been recently criticized and, despite many successful replications, the UTE, which states that unconscious thought improves decision making in complex problem situations, does not always replicate (e.g., Acker, 2008; Calvillo and Penaloza, 2009; Rey et al., 2009; Newell and Rakow, 2011). According to Strick et al. (2011), the debate concerning the UTE focuses on three main open questions: First, how stable and replicable is the UTE? Second, which boundary conditions are necessary for the UTE to appear? And third, what are the cognitive processes underlying periods of unconscious thought (Damian and Sherman, 2013; Dijkstra et al., 2013; Abadie et al., 2016, 2017)? Not neglecting the first two, the present work primarily addresses the third question. We were interested in participants’ thoughts – unconscious as well as conscious – during distraction periods and intended to shed light on the question of which cognitive processes foster decision making or attitude formation within a standard UTT paradigm.

Schooler and Melcher (1995) suggested that incubation phases, that is phases during which a pending creative or complex problem is put aside to work on something else, cause a change of people’s “mental sets”: By doing so, one may get a fresh look at the situation, which eventually results in better problem solving. This view implicates a passive process of unconscious thought as something that “just happens” while a person is working on a different task. Dijksterhuis (2004) suggested that unconscious thought is rather active, as it renders mental representations more polarized as well as better organized and clustered. However, research on the UTE has been mostly *output-centered* so far. Measures such as choices, evaluations or attribute-memories have been the variables of interest used to draw conclusions concerning the cognitive processes during presumed unconscious-thought periods. For a long time, *in-the-moment* thought processes leading to specific manifestations of such output variables have been neglected, probably because they are difficult to assess with standard cognitive methods, making it challenging to directly address the third main question (see above) raised by Strick et al. (2011). To overcome this problem, current *mind-wandering* research has been applying experience-sampling methods, which have been shown to be a valid instrument for the assessment of participants’ thought contents during all sorts of tasks (see Smallwood and Schooler, 2015 for an overview). Therefore, we argue that UTT research can benefit from the employment of such methods as they have the potential to offer a “fresh look” at the cognitive processes underlying unconscious- as well as conscious-thought periods.

Mind wandering can be described as disengaged or decoupled task-attention and has been intensively studied in recent years (Smallwood and Schooler, 2006; Mason et al., 2007). Although handling many daily tasks requires our focused attention, it is a well know phenomenon that our thoughts drift off from time to time. Even though performance on the task at hand often suffers (e.g., Mrazek et al., 2012; Rummel and Boywitt, 2014), drifting thoughts also seem to be of adaptive value (Mooneyham and Schooler, 2013; Smallwood and Andrews-Hanna, 2013). For example, mind wandering toward unsolved problems or tasks may be beneficial for problem solution or task fulfillment (Baars, 2010; Steindorf and Rummel, 2017). Mind wandering is typically measured via self-reports. So-called *thought probes* interrupt ongoing tasks and ask participants to briefly describe and/or classify their current thoughts. Responses to these probes have proven to be valid (McVay and Kane, 2012) and to be good estimates of mind-wandering frequency as they do not rely on thought-awareness (Smallwood and Schooler, 2006). Finally, they are also often found to correlate with retrospective thought questionnaires (e.g., Steindorf and Rummel, 2020), which are a similar, yet distinct mind-wandering assessment method. In such questionnaires, after task completion, participants are asked to categorize the entirety of thoughts they had experienced while working on the task into several categories. In the present experiments, online as well as retrospective mind-wandering self-reports were employed to measure and quantify thought-contents occurring within a standard UTT paradigm.

Furthermore, we considered wandering thoughts as an alternative explanation for UTEs. Previous research concerning the underlying cognitive processes of complex-problem incubation suggests that either unconscious processes or short retrieval intervals during the incubation task foster post-incubation performance (Damian and Sherman, 2013; Dijkstra et al., 2013; Abadie et al., 2016, 2017; see also Sio and Ormerod, 2009). Automatically occurring, drifting thoughts concerning a still pending problem might represent such short retrieval intervals (cf. Steindorf and Rummel, 2017): Considering a typical UTT paradigm, one might argue that during the presentation of object-attribute combinations, an unsolved problem is activated within a participant’s cognitive system (Watkins, 2008). This activation might lead to an increase in mental occupation with – or mind wandering toward – the problem during a period of distraction (Zeigarnik, 1927). Since mind wandering might foster problem solving (Baars, 2010), wandering apartment-thoughts during a distraction period might explain why problem-solving performance is improved for distracted participants. Moreover, the idea that too much deliberation can have destructive effects (Waroquier et al., 2010) as a current problem might be “thought to pieces” led us to assume that mind-wandering episodes during distraction tasks might offer just the right amount of necessary problem engagement.

Further support for our assumptions comes from the combination of findings that, during more demanding tasks, lower levels of mind wandering are reported (e.g., Rummel and Boywitt, 2014) and that more demanding distraction tasks within UTT paradigms often lead to worse problem solving compared to less demanding tasks (McMahon et al., 2011; Strick et al., 2011; Abadie et al., 2013; Waroquier et al., 2014). Focusing only on the latter finding, Strick et al. (2011) conclude that distraction tasks with high demands might compete with unconscious thoughts for resources. A different conclusion might refer to mind-wandering processes. That is, as high-demanding tasks do not leave a lot of room for mind wandering to occur, we assume that without this engagement in productive mind wandering toward the active problem, the benefit of a distraction-task period is reduced. In other words, high demands might compete with adaptive mind-wandering processes for attentional resources.

Whether high demands compete with unconscious-thought or with mind-wandering processes, both lines of argumentation suggest that during distraction tasks we allocate attentional resources toward a second ongoing cognitive process, contradicting the definition of unconscious thought as being *deliberation-without-attention* (Dijksterhuis et al., 2006). Waroquier et al. (2014) offer a solution for this dilemma by considering that attention and consciousness are not identical to each other. One can allocate cognitive resources toward the processing of specific information (attention) without being aware of the process (consciousness, or *meta-awareness* as it is often termed in the mind-wandering literature). For this reason, Strick et al. (2011) suggested replacing the term *deliberation-without-attention* with the term *deliberation-without-consciousness*. Concerning mind wandering, it is found that off-task thoughts occur both with and without awareness (Smallwood and Schooler, 2006). Sometimes we know and “feel” that our minds are drifting off, sometimes we might realize that we have been pondering tonight’s dinner plans only when being asked about our current thoughts. However, although at times we are not aware of our thoughts at the exact moment they are occupying our minds, we are able to put these thoughts into words later, suggesting that we have nevertheless allocated attentional resources toward them. During distraction-task periods within UTT experiments, aware as well as unaware cognitive processes could foster problem solving. Our thoughts might wander toward the still active, unsolved problem, with or without awareness. Attention-demanding wandering thoughts, which we are *not* aware of, might be what is referred to as “unconscious” within UTT. In the present work, using self-report methods, we intended to bring wandering thoughts into awareness by directly asking participants about their current thought processes. Especially thought probes, which do not rely on thought awareness, could reveal themselves as a promising method for gaining insight into the actual attentional processes occurring during distraction-task periods. That is, participants might experience a compound of aware and unaware problem-related mind wandering, which we intend to capture and to relate to problem-solving abilities.

In the following sections, we describe three experiments, in which we hypothesize UTEs to be mirrored by changes in mind-wandering behavior. More precisely, we expected the amount of apartment-thoughts during distraction-task incubation phases (i.e., typical unconscious-thought phases) to be related to post-incubation performance. In the first experiment, we relied on retrospective mind-wandering questionnaires for a first insight into participants’ thought processes during periods of distraction and conscious thought. In the second experiment, additional thought probes were employed to capture in-the-moment thoughts including unaware processes. The third experiment was conducted to take a closer look at awareness processes in UTT paradigms including thought probes. We first describe our general methods and plan of analyses before attending to the respective experiments.

## General Methods and Plan of Analyses

In all following *Methods* and *Results* sections, we report how we determined our sample sizes and all data exclusions, manipulations, and measures in the study (Simmons et al., 2012). Following the recommendations of Seli et al. (2018), we conceptualized mind wandering as task-unrelated thought and explained the concept to our participants accordingly. We named experimental conditions in which participants were instructed to think about previously presented objects during a filler interval *conscious thought conditions*. Experimental conditions in which participants worked on a distraction task during a filler interval were named *unconscious thought conditions*. These labels refer to the standard thought-mode manipulations from the UTT literature and do not imply participants’ actual mode of thought, as the latter represents a to-be-examined variable in the reported experiments. Our data are available under https://osf.io/4375q/ (doi: 10.17605/OSF.IO/4375Q).

### Instruments

#### Apartment Task

In all three experiments, we used a German version of an apartment task^1^ originally developed by Dijksterhuis (2004) to assess participants’ problem solving abilities in situations with high information load. Participants of this task are presented with information about four apartments. Imagining being on apartment hunt, they are supposed to familiarize themselves with and to visualize all apartments so that they will later be able to choose the best one. Each apartment is characterized by twelve attributes in total. The objectively best apartment is described by eight positive (e.g., “Apartment B has a balcony.”) and four negative (e.g., “Apartment B does not have a washing machine.”) attributes. The objectively worst apartment is described by eight negative and four positive attributes. The remaining two neutral apartments are described by six positive and six negative attributes each. For each apartment, attributes are assigned randomly from a list of twelve positive and twelve negative attributes with the only restriction being the number of positive/negative attributes. Apartment characteristics that are most essential in apartment-hunt situations (rental cost, apartment size, etc.) are not considered in the attribute list, so that they cannot overshadow other, intermediately essential, characteristics. The apartment task is typically divided into two phases, namely a *presentation* and an *evaluation* phase, which are separated by a filler interval. In the present studies’ presentation phases, the 48 apartment-attribute combinations were displayed sequentially and randomly intermixed for four seconds each, resulting in a total presentation time of 192 sec. In the later evaluation phases, participants were asked to indicate their attitude toward each of the apartments on a scale from one (*extremely negative*) to ten (*extremely positive*).

#### Filler Interval Activity

In the present experiments, two versions of the *n*-back task were used as distraction tasks within the filler interval between the apartment task’s two phases. In the *n*-back task, single letters are displayed consecutively. Participants of this task are supposed to press one key when the currently presented letter matches the one presented *n* trials earlier (target trials). For all other letters (non-target trials), they are supposed to press another key.

For the present implementations of this task, 20 different letters (*B C D F G H J K L M N P Q R S T V W Y Z*) were used and presented for 500 ms each in the center of the screen with a 300-ms inter-stimulus interval. Participants always performed one block of the *n*-back task consisting of 32 non-target and 16 target trials. The *B*-key was used as the response key for non-targets and was labeled with a red sticker. The green-labeled *C*-key was used as the target key. In the conditions with demanding distraction *n* equaled three, resulting in more letters to be constantly monitored compared to the conditions with undemanding distraction, in which *n* equaled one. Including one short introduction screen, the distraction task lasted approximately three min. Instead of working on a distraction task, the participants in the conscious thought conditions were asked to consciously think about their attitudes toward four previously presented apartments for 3 min during the filler interval.

#### Retrospective Thought Assessment

To assess the amounts of task-related and task-unrelated thoughts during the filler interval, participants of all three experiments were asked to retrospectively categorize the entirety of thoughts they had experienced during this interval into several categories using percentage scores. Participants in the conscious-thought conditions were given two response categories: (1) thoughts about the apartment task and (2) thoughts about something completely unrelated. In addition to these categories, participants in all other conditions, that is conditions including an *n*-back distraction-task, were asked to indicate the percentage of (3) thoughts about the distraction task and (4) thoughts about their performance on the distraction task. For all participants, category (2) corresponded to general off-task thoughts and was explained accordingly using multiple examples. For conscious-thought participants, category (1) corresponded to on-task thoughts. For all other participants, category (1) corresponded to a special kind of off-task thoughts and category (3) to on-task thoughts. Category (4) described task-related interferences.

### General Procedure

**FIGURE 1:**
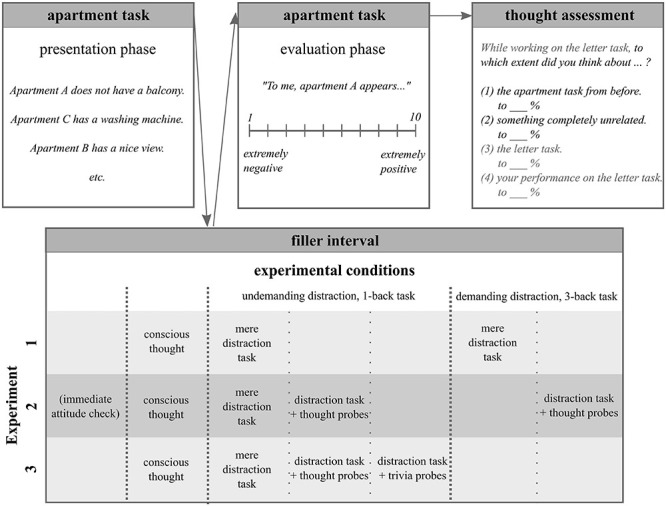
Experimental procedure. All participants worked on the apartment task’s presentation phase and evaluation phase, with both phases being separated by a filler interval. The filler interval activity varied between experimental conditions and experiments. After evaluating the apartments, participants filled in a retrospective thought-assessment questionnaire, which related to their thoughts during the filler interval. Participants in the immediate evaluation condition (Experiment 2) made an exception to this general procedure, as they worked on both apartment task phases one after the other without a filler interval.

The general procedure (excluding instructions and practice) is illustrated in Figure 1. At the beginning of each experiment, participants signed a consent form and provided demographic information. Afterward, all participants received instructions for a distraction task they would have to perform later. They were then presented with the to-be evaluated apartments. The apartment task’s presentation phase was followed by a filler interval that differed between conditions and experiments. Next, participants indicated their attitudes toward the previously presented apartments in the evaluation phase. Finally, participants’ thought contents during the filler interval were retrospectively assessed, before participants were debriefed and dismissed. Detailed procedure descriptions for each experiment are provided below.

### General Plan of Analyses

In all three reported experiments, we realized a conscious thought and several unconscious thought conditions, which differed with regard to the filler interval activities and thought assessment methods. Filler interval activities differed regarding their difficulty and in some conditions, participants’ current thoughts were probed during the task. In all conditions, retrospective thought reports were (additionally) employed after the task.

We employed a consistent plan of analyses for all experiments regarding the main dependent variables, which were apartment-task performance, amounts of apartment-related thoughts during the filler interval, and amounts of task-unrelated thoughts during the filler interval. We always first ran a one-factorial ANOVA with the experimental condition as fixed factor testing for overall group differences. We then conducted follow-up analyses using Helmert contrasts, for which the first contrast always tested for an overall UTE and the following contrast(s) for the experiment-specific manipulations within the unconscious thought conditions. When necessary, we finally conducted additional simple comparisons to further disentangle significant effects.

Since we worked with experimental designs, the focus of our analyses was on group differences. Full correlation tables for each experimental condition from Experiments 1 to 3 as well as from a joint data set (see below) can be found in our Supplementary Material under https://osf.io/4375q/ (doi: 10.17605/OSF.IO/4375Q).

### General Measures

The performance on the distraction task (*n*-back) was defined in terms of the sensitivity index *d’*. The performance on the apartment task was defined as the difference in the subjective attitude values between the objectively best and worst apartment (see for example Dijksterhuis, 2004; Dijksterhuis and Nordgren, 2006). Higher values thus represent a better performance. The amounts of retrospectively reported task-unrelated and apartment-related thought during the filler interval were specified using percentage scores. Thoughts that were completely unrelated to any of the study’s tasks and thus corresponded to response category (2) were considered as *task-unrelated thoughts* (TUTs) in all analyses. Thoughts that were related to the apartment task [response category (1)] were considered as *apartment thoughts* (ATs).^2^ Thoughts about the distraction task itself and the performance on this task are both distraction-task related. Because they directly result from TUTs and ATs [%_distraction – task related thoughts_ = 100% – (%_TUTs_ + %_ATs_)] and because the latter two were our variables of interest, we only analyzed TUTs and ATs.

## Experiment 1

We ran the first experiment to establish the apartment-task paradigm and to gain initial insights into thought processes during conscious and unconscious thought filler intervals. For this purpose, we employed a standard UTT experiment, in which one group of participants was supposed to consciously think about previously presented apartments before evaluating them. Two other groups worked on a distraction task instead during the filler interval. To additionally examine the influence of the distraction task’s difficulty on thought reports as well as the apartment-task performance, we employed both an undemanding and a demanding version of the distraction task. In Experiment 1, retrospective thought reports were supposed to provide insights into participant’s cognitive processes leading to the solution of the apartment problem.

### Method

#### Participants and Design

An *a priori* power analysis with the software G^∗^Power (Faul et al., 2007) was conducted to determine the required sample size for the planned contrast analysis central to our hypotheses that would allow to reveal medium-size effects with an α = 5% and 1-β = 80%. This analysis suggested a required sample-size of at least *N* = 128. To cover for potential drop-outs, we tested a total of 153 participants at Heidelberg University, Germany in groups no larger than six. Data of five participants were excluded due to poor performance on the distraction task (*d*’ < 0). Comparing these participants’ performances to the non-excluded participants’ good performances (mean *d’* = 1.99 for the demanding distraction task, mean *d’* = 3.00 for the undemanding distraction task), we assumed that the excluded participants did not pay sufficient attention to the distraction task, potentially changing their mind-wandering behavior and/or influencing possible unconscious thought processes. Another three participants’ data were excluded due to missing values on at least one of the dependent variables of interest. Missing values resulted from participants not properly filling out the though-assessment questionnaire or the apartment evaluation. Analyses were thus executed with *N* = 145 (*M*_age_ = 21.82, *SD*_age_ = 3.96; 123 female). We employed a one-factorial design with *thought condition* being manipulated between participants: conscious thought (*n* = 48), unconscious thought with demanding distraction (*n* = 49), and unconscious thought with undemanding distraction (*n* = 48).

#### Procedure

The three experimental conditions differed regarding the filler interval activity. Accordingly, at the beginning of the experiment, the participants in the unconscious thought condition with demanding distraction received instructions for the 3-back task and were presented with practice trials. The participants in the unconscious thought condition with undemanding distraction and in the conscious thought condition (to keep the procedure equal for all conditions) read instructions for and practiced the 1-back task. Having finished the practice trials, all participants were told that they would later work on more trials of the task. For the current moment, however, they would work on a different task. All participants were instructed regarding the apartment task and the presentation of apartment-attribute combinations started. After the apartment task’s presentation phase, the participants in both unconscious thought conditions worked on the respective version of the distraction task while participants in the conscious thought condition were asked to actively think about their attitude concerning all previously presented apartments. Then, in the evaluation phase of the apartment task, all participants indicated their attitude toward each of the presented apartments. Finally, they filled out the retrospective thought-assessment questionnaire.

### Results

#### Distraction-Task Performance

The performance in the condition with a demanding distraction task (3-back, *M* = 1.99, *SD* = 0.70) was significantly worse than the performance in the condition with an undemanding distraction task (1-back, *M* = 3.00, *SD* = 0.66), *t*(95) = 7.37, *p* < 0.001, *d* = 1.50, reflecting the fact that the demanding task was more difficult than the undemanding one.

#### Apartment-Task Performance

**FIGURE 2:**
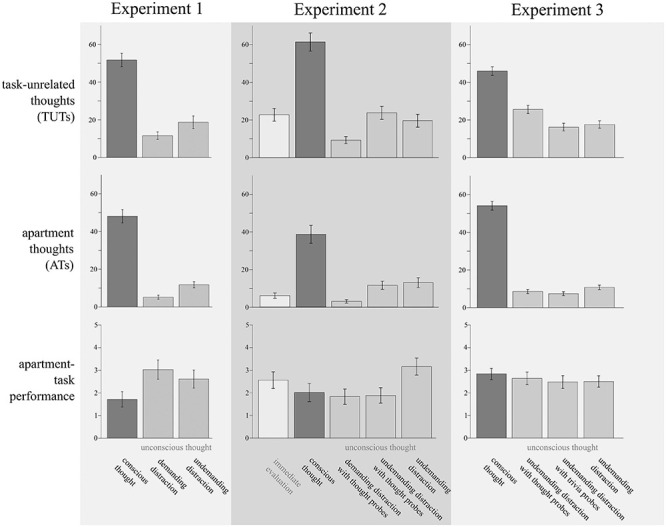
Descriptive data for the main analyses. Columns represent the respective experiment, rows the respective variable. The bars’ colors stand for the thought-mode manipulations employed, with darker gray representing conscious-thought manipulations and lighter gray unconscious-thought manipulations. The immediate-evaluation condition of Experiment 2 is displayed in white. Patterns of results are explained and discussed within the running text. Error bars represent standard errors of the means.

As illustrated in Figure 2, there was a marginally significant difference between the three experimental conditions regarding the apartment-task performance, *F*(2, 142) = 2.96, *p* = 0.055, η^2^*_p_* = 0.04. Helmert contrasts indicated an UTE. That is, performance was generally worse in the conscious thought condition compared to the two unconscious thought conditions, *F*(1, 142) = 5.33, *p* = 0.022. Between the two unconscious thought conditions, the apartment-task performance did not differ, *F*(1, 142) = 0.57, *p* = 0.451. Thus, employing a distraction task during the filler interval, regardless of this task’s difficulty, fostered participants’ problem solving abilities in comparison to those participants who actively thought about the problem during the filler interval.

#### Retrospective Thought Reports

The amount of TUTs (see Figure 2) differed significantly between the three experimental conditions, *F*(2, 142) = 50.58, *p* < 0.001, η^2^*_p_* = 0.42. Helmert contrasts showed that the percentage of TUTs in the conscious thought condition was generally higher than in the two unconscious thought conditions, *F*(1, 142) = 98.17, *p* < 0.001. Between the two unconscious thought conditions the percentage of TUTs only differed marginally, *F*(1, 142) = 2.78, *p* = 0.097, with a numerically higher number in the condition with undemanding distraction (cf. Rummel and Boywitt, 2014). The amount of ATs (see Figure 2) also varied with experimental conditions, *F*(2, 142) = 99.59, *p* < 0.001, η^2^*_p_* = 0.58. Helmert contrasts indicated that there were more ATs in the conscious thought condition than in the two unconscious thought conditions, *F*(1, 142) = 194.80, *p* < 0.001. The percentage of ATs in the undemanding unconscious thought condition was still higher than in the demanding unconscious thought condition, *F*(1, 142) = 4.04, *p* = 0.046. That is, participants in the conscious thought condition thought about the apartments for roughly half of the time. During the other half, they thought about unrelated matters. Participants in both unconscious thought conditions spent the majority of their filler interval time thinking about the distraction task. However, there was still room for TUTs and ATs, especially for participants working on the undemanding task.

### Discussion

In Experiment 1, we established the apartment task paradigm producing an UTE. This effect was independent of the distraction task’s difficulty, although thought patterns differed between both unconscious thought groups. Overall, distracted participants reported lower levels of ATs and performed better on the apartment task. Moreover, conscious-thought participants, who showed the highest levels of apartment thought, performed worse on the apartment task. Using mind-wandering assessment methods, we were able to demonstrate that a distraction task does not leave a lot of room for deliberation about the apartment-task problem. Hinting toward a competition for attentional resources of task-related and adaptive-mind-wandering processes, we found the lowest levels of ATs for participants in the demanding distraction condition. We had additionally expected worse problem-solving performance for participants in this condition as a result of this competition. However, participants of the demanding condition performed comparably well as those of the undemanding condition on the apartment task, challenging our assumptions about apartment-related mind wandering being an alternative explanation for the UTE. Still, because all participants working on any kind of a distraction task showed lower levels of ATs as well as a better apartment task performance than conscious-thought participants, the possibility remains that the unconscious thought conditions fostered “just the right amount” of ATs, which may be necessary and sufficient for a good apartment task performance.

Participants in the conscious thought condition indicated high levels of general TUTs. They only spent about half of the filler-interval time thinking about the apartments, which was their actual task. This finding might indicate that the filler interval was too long, so that participants had too much time and “thought the apartment problem to pieces.” Thus, a possible alternative explanation for our findings might be that unconscious thoughts, or fewer apartment thoughts, do not generally lead to better evaluations. Rather, too intensive conscious thought about the apartment problem may have had destructive effects on the apartment task solution.

## Experiment 2

In the second experiment, we included an additional baseline condition to be better able to interpret the effects of conscious thought manipulations. In this condition, participants did not have time to consciously (or unconsciously) think about the previously presented apartments before evaluating them. The objective was to investigate whether a high amount of ATs in the conscious thought condition would result in poorer apartment-task performance due to *overthinking* compared to no ATs in a condition without a filler interval.

Apart from including this new condition, the structure of Experiment 2 resembled the first experiment’s structure with unconscious thought conditions differing in task demands for the distraction task. Additionally, to examine thought processes in the exact moment they are happening and to capture possible unaware processes, we employed online thought probes during the filler interval. As stated in the introduction section, thought probes are frequently used in mind-wandering experiments and interrupt participants who are working on a task by asking them to briefly describe and/or classify their current thoughts’ content. Another advantage is that they do not rely on thought awareness (Smallwood and Schooler, 2006), so that they might be able to capture thought processes that participants are unaware of, which possibly are not captured as well by retrospective thought reports. Thought probes proved to be reasonably valid mind-wandering indicators (e.g., McVay and Kane, 2012) and do not interfere with performance on ongoing cognitive tasks (Wiemers and Redick, 2019). Yet, it may well be that asking participants to report on their thoughts during the filler interval might disrupt unconscious thought processes. For this purpose, we additionally employed a condition without such task-interruptions to control for possible reactive effects on apartment-task performance. Furthermore, such a condition ensures comparability with regard to Experiment 1.

### Method

#### Participants and Design

In groups of up to six, we tested 152 participants at Heidelberg University, Germany, and 162 participants at Mannheim University^3^, Germany, ensuring that participants had not participated in Experiment 1 and that group sizes were comparable to those in Experiment 1. Data of seven participants were discarded due to poor performance (*d’* < 0) in the distraction task (to compare, means for the non-excluded participants, *d’* = 2.14 for the demanding distraction task, *d’* = 2.89 for the undemanding distraction task). Another four participants’ data were excluded due to missing values on at least one of the dependent variables of interest. Analyses were thus executed with *N* = 303 (*M*_age_ = 22.78, *SD*_age_ = 4.06; 219 female). We employed a one-factorial design with the *thought condition* being manipulated between participants [immediate evaluation (*n* = 59), conscious thought (*n* = 61), and three unconscious thought conditions: demanding distraction with thought probes (*n* = 60), undemanding distraction with thought probes (*n* = 60), undemanding distraction without thought probes (*n* = 63)].

#### Procedure

As in Experiment 1, we employed the procedure outlined in the General Procedure section with the experimental conditions differing regarding the filler interval activity. We used a *1-back* task as the filler interval distraction task for the two unconscious thought conditions with undemanding distraction and a *3-back* task for the unconscious thought condition with demanding distraction. While working on these tasks, the participants in the conditions including thought probes were interrupted after each sequence of 12 trials (resulting in a total of four thought probes) and asked about their current thoughts. They were supposed to categorize their current thoughts’ content as being *n*-back-task-related, related to their performance on the *n*-back task, related to the apartment task, or unrelated to any task in the current study. Participants in the conscious thought condition were supposed to actively think about their attitude toward all previously presented apartments during the filler interval.

Having been presented with distraction-task instructions, the apartment task’s presentation phase, the filler interval activity, and the apartment task’s evaluation phase in this order, participants were asked about their thoughts during the filler interval. We employed the same retrospective thought-assessment questionnaire as in Experiment 1, but for Heidelberg participants only. We decided to include this questionnaire on short notice at a time point at which data collection was already ongoing in Mannheim. The participants in the conscious thought condition filled out the version with two response options, that is, (1) thoughts about the apartment task, and (2) thoughts about something completely unrelated. The participants in all other conditions filled out the version with four response options, that is, (1), (2) as well as (3) thoughts about the distraction task, and (4) thoughts about their performance on the distraction task.

The participants in the immediate-evaluation condition worked on the individual parts of the experiment in a different order. They were asked to indicate their attitude toward each of the presented apartments directly after the presentation phase of the apartment task. That is, for these participants there was no filler interval between the apartment task’s two phases. After completing the evaluation phase, they worked on the 1-back version of the *n*-back task including thought probes and filled in the retrospective thought-assessment questionnaire afterward.

### Results

As for Experiment 1, we ran an ANOVA for each of our dependent variables of interest to test for overall group-differences between the experimental conditions. The performance on the distraction task varied between the four experimental conditions in which participants worked on the n-back filler task, *F*(3, 238) = 15.55, *p* < 0.001, η^2^*_p_* = 0.16. All other ANOVAs included all five experimental conditions. The performance on the apartment task (see Figure 2) also varied with experimental conditions, *F*(4, 298) = 2.43, *p* = 0.048, η^2^*_p_* = 0.03. Because we had retrospective thought data available for (most of) the Heidelberg participants only, we ran the analyses concerning retrospectively reported TUTs and ATs (see Figure 2) with *N* = 144. The amount of retrospectively reported TUTs differed significantly between the experimental conditions, *F*(4, 139) = 31.63, *p* < 0.001, η^2^*_p_* = 0.48, as did the amount of retrospectively reported ATs, *F*(4, 139) = 25.72, *p* < 0.001, η^2^*_p_* = 0.43.

The amounts of online reported TUTs and ATs were defined as the sum of thought probes in which participants self-categorized their thoughts as being task-unrelated or apartment-related, respectively. Online reports of TUTs and ATs were collected in three conditions only (immediate evaluation, unconscious thought with undemanding distraction and thought probes, and unconscious thought with demanding distraction and thought probes). Online reported TUTs varied between groups, *F*(2, 176) = 16.67, *p* < 0.001, η^2^*_p_* = 0.16, as did online reported ATs, *F*(2, 176) = 5.86, *p* = 0.003, η^2^*_p_* = 0.06.

In the following sections, we report planned contrasts that were carried out based on the above reported ANOVAs.

#### Immediate Evaluation Condition

For comparability (between experiments) and clarity reasons, we prepend reporting all planned contrasts, which include the immediate evaluation condition as a reference condition. Our main objective for including this condition was to test whether a large amount of ATs in the conscious thought condition would result in poorer apartment-task performance due to overthinking compared to no ATs in the immediate evaluation condition. A first planned contrast showed, however, that the immediate evaluation and the conscious thought condition achieved a comparable apartment-task performance, *F*(1, 298) = 1.09, *p* = 0.298. Furthermore, within following contrasts, we found that there were no significant differences between each of the unconscious thought conditions and the immediate evaluation condition regarding the apartment-task performance, all *F*s ≤ 1.93, all *p*s ≥ 0.166. That is, a 3-min distraction interval within the apartment task paradigm did not result in better apartment evaluations compared to evaluations submitted right after apartment presentation.

Next, we contrasted the immediate evaluation condition with the unconscious thought conditions with undemanding distraction, because participants in these conditions worked on the same task (1-back) as immediate-evaluation participants. Concerning filler-interval measures, we did not find significant differences regarding distraction-task performance, all *F*s ≤ 0.92, all *p*s ≥ 0.338. We also did not find any significant differences regarding online, *F*(1, 176) = 0.86, *p* = 0.356, and retrospective TUTs (all *F*s < 0.40, all *p*s ≥ 0.530) as well as retrospective ATs (all *F*s ≤ 3.11, all *p*s ≥ 0.080). However, thought-probe results indicated that there were more online-reported ATs, *F*(1, 176) = 7.53, *p* = 0.007, in the unconscious thought condition with undemanding distraction (*M* = 0.48, *SD* = 0.68) than in the immediate evaluation condition (*M* = 0.22, *SD* = 0.42). Participants who performed the distraction task after the apartment task was finished showed fewer ATs than participants who performed it before they made their apartment judgments. This pattern could be interpreted as a *Zeigarnik-like* effect (Zeigarnik, 1927).

All in all, besides this Zeigarnik-like effect, there were no significant differences between participants in the immediate evaluation condition and participants in the conditions featuring a delayed evaluation of apartments. As stated above, we next specified planned contrasts comparing the delayed evaluation conditions, analogously to the analyses conducted in Experiment 1. The immediate evaluation condition was always weighted with zero for these analyses.

#### Distraction-Task Performance

Helmert contrasts revealed that the performance in the unconscious thought condition with a demanding (3-back) distraction task (*M* = 2.14, *SD* = 0.86) differed significantly from the performance in the two unconscious thought conditions with an undemanding (1-back) distraction task, *F*(1, 238) = 37.59, *p* < 0.001. Performance between the undemanding-distraction condition without thought probes (*M* = 2.90, *SD* = 0.68) and that with thought probes (*M* = 2.82, *SD* = 0.76) did not differ, *F*(1, 238) = 0.34, *p* = 0.560. As in Experiment 1, performance on the demanding distraction task was worse than on the undemanding one. The presence of thought probes did not affect the distraction task performance.

#### Apartment-Task Performance

The first Helmert contrast (conscious thought versus the three unconscious thought conditions) did not indicate an UTE, *F*(1, 298) = 0.43, *p* = 0.514. That is, participants who performed a distraction task during the filler interval did not generally perform better than participants who consciously thought about the apartment problem during the filler interval. Further contrasting the three unconscious thought conditions with each other, we found participants who worked on the demanding version of the distraction task to achieve a similar apartment-task performance as participants who worked on the undemanding distraction task, *F*(1, 298) = 2.35, *p* = 0.127. When contrasting both undemanding distraction conditions, we found participants who were not probed while working on the distraction task to performed better than those who were probed, *F*(1, 298) = 6.16, *p* = 0.014. Finally, employing further simple planned contrasts to compare the conscious thought condition with each of the unconscious-thought conditions, we found an UTE for the condition with undemanding distraction without thought probes only, *F*(1, 298) = 4.98, *p* = 0.026, both other *F*s ≤ 0.13 and *p*s ≥ 0.724. This pattern suggests that unconscious thought participants whose thoughts were not probed during the filler interval found better solutions to the apartment problem than participants who consciously thought about the apartments.

#### Retrospective Thought Reports

Helmert contrasts revealed that conscious thought participants reported more TUTs than participants in the unconscious thought conditions, *F*(1, 139) = 116.17, *p* < 0.001. Participants who had worked on the demanding distraction task showed fewer TUTs than participants who had worked on the undemanding distraction task, *F*(1, 139) = 8.12, *p* = 0.005. Undemanding distraction-task participants who had received thought probes showed similar levels of TUTs as participants who did not receive any thought probes, *F*(1, 139) = 0.71, *p* = 0.401.

Regarding the amount of ATs, Helmert contrasts showed that more ATs were reported in the conscious thought condition than in the unconscious thought conditions, *F*(1, 139) = 84.77, *p* < 0.001. Participants who had performed the demanding distraction task showed fewer ATs than participants who had performed the undemanding distraction task, *F*(1, 139) = 7.30, *p* = 0.008. Moreover, undemanding-distraction participants, who had received thought probes while working on the distraction task, showed similar levels of ATs as participants who did not receive thought probes, *F*(1, 139) = 0.14, *p* = 0.710.

These findings suggest that conscious thought participants had followed our instructions to consciously think about the apartments. However, they only did so for roughly 40 percent of the time. The high demands of the 3-back task almost entirely kept participants in the demanding unconscious thought condition from thinking about the apartments and unrelated matters whereas participants in both undemanding unconscious thought conditions still thought about the apartments and other issues from time to time. Thought probes had no influence on reported thoughts.

#### Online Thought Reports

Unconscious-thought participants who had performed the demanding 3-back version of the distraction task (*M* = 0.42, *SD* = 0.59) reported significantly fewer TUTs than unconscious-thought participants who had performed the undemanding 1-back version (*M* = 1.17, *SD* = 1.04), *F*(1, 176) = 20.10, *p* < 0.001. The same pattern was found for ATs, with fewer ATs for participants who had worked on the demanding version (*M* = 0.18, *SD* = 0.43) compared to those who had worked on the undemanding version of the distraction task (*M* = 0.48, *SD* = 0.68), *F*(1, 176) = 9.88, *p* = 0.001. Overall, patterns of online thought reports mirrored retrospective thought reports patterns. Even numerically, percentages of TUTs and ATs were very similar whether measured by online or by retrospective methods^4^.

### Discussion

In Experiment 2, we found an UTE only for participants who did not receive thought probes while performing the distraction task during the filler interval. These participants performed better in the apartment task than participants who consciously thought about the apartments during the filler interval. They also retrospectively reported less apartment-related thoughts compared to conscious thought participants, replicating the findings from Experiment 1. The second experiment’s results also ruled out the possibility that conscious thought participants might overthink the apartment problem, as they did not perform worse than participants who were not given time to consciously or unconsciously think about the apartments before evaluating them.

In Experiment 2, we validated the retrospective thought reports employed in Experiment 1. Thought probes provided similar thought descriptions as retrospective questionnaires. This finding suggests that after and during task completion, participants seem to be well aware of their recent thought processes. Because thought probes were found to be valid mind-wandering-frequency indicators (McVay and Kane, 2012) which do not rely on thought awareness because they capture participants’ thoughts in the moment they are happening (Smallwood and Schooler, 2006), we conclude that we might have captured a compound of aware as well as unaware wandering thoughts. A distinction between the two kinds of mind-wandering assessment might be that retrospective reports were not intrusive, due to them capturing mind wandering after distraction-task completion, that is, without interfering with any thought processes during the task. Thought probes, however, might have altered participants’ thought experiences during the distraction task by bringing the unaware portions of the thought compound into awareness. Results obtained using the retrospective mind-wandering questionnaires should, however, be interpreted with caution. We acknowledge that the statistical power to detect effects on this measure was certainly limited because only participants from one out of two universities provided retrospective thought reports resulting in a smaller sample size for this measure compared to all other measures.

Unexpectedly, we found that the UTE disappeared when we employed thought probes during the filler interval, leading us to assume a detrimental nature of such probes to the processes producing the UTE. It has already been found that changing thought-probe characteristics within mind-wandering experiments can lead to differences in results (Seli et al., 2013; Weinstein et al., 2018; Robison et al., 2019), which made it reasonable to assume that the mere presence of thought probes might have interfered with (unconscious) thoughts for at least two reasons (Steindorf et al., 2020): One reason could be that thought probes may have interrupted the ongoing task and thereby ongoing (unconscious) thought processes. Alternatively or additionally, thought probes may have made participants more aware of their current states of thought during the distraction task, whereas an absence of thought awareness might be a necessary criterion for an UTE to appear. To test these two competing assumptions and to further replicate the negative association between AT-levels and apartment-task performance, we conducted a third experiment.

## Experiment 3

In the third experiment our aim was to, once more, replicate the UTE and its negative association with the number of ATs. For this reason, we included the same conscious thought condition as well as the same unconscious thought condition (undemanding distraction task without thought probes) as in the first and second experiments. To take a closer look at the effect of thought probes on thought processes as well as on apartment-task performance, we included one condition with an undemanding distraction task and thought probes (see Experiment 2) and one new condition in which participants were only interrupted during the undemanding distraction task, but not asked about their current thoughts. If mere interruption was responsible for the lack of an UTE when including thought probes as found in Experiment 2, the UTE should be absent in this condition. If, however, thought awareness was a necessary condition for the effect to vanish, we would expect a better apartment-task performance in this condition compared to both the condition including regular thought probes and the conscious thought condition. A lack of thought awareness as a necessary condition for the UTE would further support the assumption of a deliberation-without-consciousness effect (Strick et al., 2011). Thought probes might add consciousness/awareness to the deliberation part, diminishing its beneficial effect on problem solving performance.

### Method

#### Participants and Design

In groups of up to six, we tested 289 participants at Heidelberg University, Germany, and 108 participants at the University of Mannheim, Germany^5^. To be able to calculate correlations between AT-levels and apartment-task performance within experimental conditions, we substantially increased the group sizes for this experiment. Participants of Experiment 3 had not taken part in Experiment 1 and 2. Data of one participant were discarded due to poor performance (*d’* < 0) in the distraction task (to compare, mean for the non-excluded participants, *d’* = 3.07). Another five participants’ data were excluded because they were handed out no or a wrong retrospective thought-assessment questionnaire. Yet another 17 participants’ data were excluded due to missing values on at least one of the dependent variables of interest. Analyses were thus executed with *N* = 374 (*M*_age_ = 21.64, *SD*_age_ = 3.19; 292 female). We employed a one-factorial design with the *thought condition^6^* being manipulated between participants. We had a conscious thought (*n* = 96) and three different unconscious thought conditions with undemanding distraction: one without thought probes (*n* = 92), one with thought probes (*N* = 94), and one with trivia probes (*n* = 92).

#### Procedure

We adhered to the general procedure and implemented the differences between experimental conditions within the filler interval as follows: We employed the *1-back* version of the *n*-back task as the filler interval distraction task for all three unconscious thought conditions. The two conditions with and without thought probes were equivalent to those employed in Experiment 2. Instead of being presented with thought probes, participants in the trivia-probe condition were presented with a trivia question after each sequence of 12 trials (as was the case for the thought probes). Each trivia question had four response options, only one of which was correct [e.g., *Who wrote the fantasy novels “Lord of the Rings”? (1) John Ronald Reul Tolkien (2) Joanna Kathleen Rowling (3) Pete Johnson (4) Jeff Kinney*]. As in the previous experiments, participants in the conscious thought condition were supposed to actively think about their attitude toward all previously presented apartments during the filler interval.

Having been presented with instructions, the apartment task’s presentation phase, the filler interval activity, and the apartment task’s evaluation phase in this order, participants were asked about their thoughts during the filler interval. We employed the same retrospective thought-assessment questionnaire as in Experiments 1 and 2. The participants in the conscious thought condition filled in the version with two response options, that is, (1) thoughts about the apartment task, (2) thoughts about something completely unrelated. The participants in all other conditions filled in the version with four response options, that is, (1), (2), (3) thoughts about the distraction task, and (4) thoughts about their performance on the distraction task.

### Results

#### Distraction-Task Performance

All unconscious thought conditions featured the same 1-back distraction task in Experiment 3. As expected, there was no significant difference between the unconscious thought conditions concerning the performance on the distraction task, *F*(2, 275) = 1.26, *p* < 0.29, η^2^*_p_* = 0.009.

#### Apartment-Task Performance

There was no significant main effect of the experimental condition for the performance on the apartment task (see Figure 2), *F*(3, 370) = 0.38, *p* = 0.765, η^2^*_p_* = 0.00. Simple contrasts further indicated that performance in each unconscious thought condition was comparable to the performance in the conscious thought condition, all *F*s ≤ 0.90, all *p*s ≥ 0.343.

#### Retrospective Thought Reports

As in Experiments 1 and 2, we analyzed TUTs and ATs as reported on the retrospective questionnaires (see Figure 2). The amount of TUTs during the filler interval varied between experimental conditions, *F*(3, 370) = 41.94, *p* < 0.001, η^2^*_p_* = 0.25. The first Helmert contrast indicated that the percentage of TUTs was higher in the conscious thought condition compared to all three unconscious thought conditions, *F*(1, 370) = 114.92, *p* < 0.001. Further comparing the unconscious thought conditions with each other, we found that participants who had received thought probes reported higher levels of TUTs than participants who had received trivia probes or no thought probes, *F*(1, 370) = 11.06, *p* = 0.001^7^. The latter two conditions did not differ from each other concerning the amount of TUTs, *F*(1, 370) = 0.21, *p* = 0.649.

The amount of ATs also varied between experimental conditions, *F*(3, 370) = 228.71, *p* < 0.001, η^2^*_p_* = 0.65. Helmert contrasts indicated higher AT levels in the conscious thought compared to all other conditions, *F*(1, 370) = 683.56, *p* < 0.001, but no differences between thought-probed, trivia-probed and unprobed unconscious-thought conditions, all *F*s ≤ 2.41, all *ps* ≥ 0.122.

#### Online Thought Reports

Thought probes were employed in one condition only. Participants reported numerically similar levels of TUTs (*M* = 1.22, *SD* = 1.01) and ATs (*M* = 0.38, *SD* = 0.55) as in the corresponding condition in Experiment 2.

#### Correlational Analyses

To more directly test for a relation between ATs during the filler interval and later apartment-task performance, we correlated these measures within conditions. Retrospectively reported ATs did not correlate with the apartment-task performance in any of the experimental conditions, all *p*s ≥ 0.242. The same held for the correlation between retrospectively reported TUTs and the apartment-task performance, all *p*s ≥ 0.287, except for the unconscious thought condition without thought probes in which TUTs correlated negatively with the apartment-task performance, *r* = −0.24, *p* = 0.022. We can only speculate about the interpretation of this single significant correlation and therefore refrain from further expanding on this result.

### Discussion

In Experiment 3, we did not replicate the UTE, which we had previously found in both Experiments 1 and 2. In addition, comparisons of apartment-task performance between the unconscious thought conditions were not conclusive. We did not replicate the detrimental effect that thought probes had had in Experiment 2. Consequently, we cannot draw any conclusions concerning a lack of thought awareness as a necessary criterion for the UTE to appear. However, thought probes had an influence on retrospectively reported general TUTs, with more wandering thoughts found in participants who had been explicitly asked about their state of thought compared to participants whose thoughts had not been probed. This influence of thought probes goes beyond mere interruption, because participants who were merely interrupted did not show such increased levels of TUTs. One could speculate that the online thought probes made participants more aware of mind wandering instances during the distraction task which, in turn, resulted in higher levels of retrospectively reported TUTs.

## Joint Analysis of the Unconscious Thought Effect

Having employed the same conscious thought and the same unconscious thought (undemanding distraction without thought probes) condition in all three experiments allowed us to collapse the data for these conditions to conduct a joined analysis of the UTE with *N* = 408 (*n*_*conscious*_ = 205, *n*_*unconscious*_ = 203). We ran a 2 (experimental conditions) x 3 (experiments) ANOVA for the apartment-task performance, which revealed no significant main effect of experiment, *F*(2, 402) = 1.23, *p* = 0.295, η^2^*_p_* = 0.01, but a significant effect of experimental condition, *F*(1, 402) = 4.33, *p* = 0.038, η^2^*_p_* = 0.01, suggesting that, overall, there was an UTE: Participants in the unconscious thought condition (*M* = 2.73, *SD* = 2.69) performed better on the apartment task than participants in the conscious thought condition (*M* = 2.33, *SD* = 2.66), which equates to a small effect with a Cohen’s *d* of 0.15. Notably, the interaction was significant, *F*(2, 402) = 3.42, *p* = 0.034, η^2^*_p_* = 0.02, bolstering the cross experimental observation that the UTE was present in Experiments 1, *t*(94) = −1.71, *p* = 0.045 (one-tailed), and 2, *t*(122) = −2.08, *p* = 0.020 (one-tailed), but not in Experiment 3, *t*(186) = 0.95, *p* = 0.173 (one-tailed).

Concerning ATs, there was a significant main effect of the experimental condition, *F*(1, 337) = 240.33, *p* < 0.001, η^2^*_p_* = 0.42, but no significant main effect of the experiment, *F*(2, 337) = 2.89, *p* = 0.057, η^2^*_p_* = 0.02, as revealed by a 2 × 3 ANOVA. On average, participants in the conscious-thought conditions (*M* = 49.84, *SD* = 24.18) reported having experienced more ATs than participants in the unconscious-thought conditions (*M* = 11.46, *SD* = 11.89). Notably, as for the apartment-task performance, there was a significant interaction, *F*(2, 337) = 5.15, *p* = 0.006, η^2^*_p_* = 0.03. This interaction did, however, not concern the presence or absence of a significant difference in the amount of ATs between the conscious- and the unconscious-thought conditions, but the extent of this difference.

Concerning TUTs, we also ran a 2 (experimental conditions) × 3 (experiments) ANOVA. There was a significant main effect of the experimental condition, *F*(1, 337) = 168.57, *p* < 0.001, η^2^*_p_* = 0.33, and of the experiment factor, *F*(2, 337) = 3.71, *p* = 0.026, η^2^*_p_* = 0.02. The interaction remained non-significant, *F*(2, 337) = 2.14, *p* = 0.119, η^2^*_p_* = 0.01. On average, participants in the conscious-thought conditions (*M* = 50.10, *SD* = 24.14) reported having experienced more ATs than participants in the unconscious-thought conditions (*M* = 18.26, *SD* = 20.01). Average TUT levels also varied significantly between experiments (M_Experiment 1_ = 36.20, SD_Experiment 1_ = 28.82; M_Experiment 2_ = 40.15, SD_Experiment 2_ = 30.66; M_Experiment 3_ = 32.05, SD_Experiment 3_ = 25.16).

Within-condition Pearson correlations between all measures of interest for the joint data set are provided in the Supplementary Materials under https://osf.io/4375q/ (doi: 10.17605/OSF.IO/4375Q). As none of the bivariate correlations was significant, we did not conduct any further correlation or mediation analyses.

### Discussion

In this joint analysis of the UTE, we found that participants in an unconscious thought condition performed better on the apartment-evaluation task than participants in a conscious thought condition. However, this effect was of a small size only. This finding is in line with a meta-analysis by Strick et al. (2011), which supports the existence of the UTE as a small effect. However, our sample sizes in the single experiments were based on a medium-sized effect, thus hinting toward power issues and advising caution when interpreting (especially null-) effects. Even though we tested as many as 822 participants overall, and included more participants per condition than most of the previous studies on the UTE, sample sizes should have been even larger to identify small effects in the present data. This especially holds for the analyses of the thought reports for Experiment 2 which only participants from one out of two universities provided. However, sample sizes for the detection of small effects may not easily be achieved in laboratory studies and running experiments online comes with additional caveats regarding data quality (Chmielewski and Kucker, 2020). Furthermore, it is debatable to which extent small effects would be of practical relevance even when they are detected with sufficient power. Consequently, we find the present results worth to be discussed although we acknowledge that the statistical power to detect small-sized effects was certainly limited.

In the joint analyses, we also found that participants in the conscious-thought conditions of all three experiments experienced more ATs (and more TUTs) compared to participants in the unconscious-thought conditions with undemanding distraction and without thought probes. These results qualify as a check that the manipulation of conscious versus unconscious thought was successful. Additionally, however, these results represent a critical test of the UTT’s fundamental assumptions. Participants who were instructed to consciously think about the apartments actually did so, even though they spent about 50 percent of the time thinking about unrelated things. Thus, the filler interval did not seem to represent an episode of uninterruptedly ongoing conscious apartment-thought. Participants in the unconscious-thought conditions spent significantly less time thinking about the apartments. Still, on average (as found in the joint analysis), they experienced ATs for a little more than 10 percent of their distraction time. Thus, the filler interval in the unconscious thought condition did not seem to represent *pure* unconscious thought. Of course, as proposed in the UTT, there might still be unconscious thought at play. How both types of thought relate to the apartment-task performance, cannot be inferred from our results. However, it is still an interesting finding that a larger amount of ATs (conscious condition) does not improve the apartment-task performance. This finding implies that this kind of conscious thought does indeed not seem to be helpful when it comes to complex decisions supporting one of the fundamental assumptions of the UTT.

Notably, in our joint analyses of the apartment-task performance and thought contents, there were significant main effects of and interactions with the experiment factor outlining a notable degree of variability concerning the observed effects and their sizes. Especially when it comes to the apartment-task-performance measure, our study’s three experiments represent a successful as well as a failed (see also Acker, 2008; Calvillo and Penaloza, 2009; Rey et al., 2009; Newell and Rakow, 2011) replication attempt of the UTE (for similar meta-analytic findings, see also Nieuwenstein et al., 2015).

## General Discussion

### Unconscious Thought Effect

Making tough decisions without any cognitive effort sounds like a good deal. Because of this obvious appeal, the UTT has been extensively studied. A meta-analysis by Strick et al. (2011) identified the UTE as a real effect, which is, however, moderated by many factors such as distraction-task features, problem-presentation features, and filler-interval length. A later meta-analysis by Nieuwenstein et al. (2015) heavily criticized the UTT, stating that there exists no support for its notions. Adding to this ongoing discussion, we ran three experiments with the goal to get a fresh look at the UTE and its underlying cognitive processes. Overall, in our joined analysis, we found an UTE of a small effect size. However, even though we employed similar methods in all three experiments, our findings were still mixed. For all experiments, we used the same materials, and we collected three independent samples with quite similar characteristics (German university students). An UTE was present in Experiments 1 and 2, but absent in Experiment 3. The possibility remains that due to Type II errors this is a natural occurrence when running nearly the same experiment repeatedly. We also want to acknowledge that we had some difficulties collecting enough “naive” participants for the third study as most members of our participant pool had already participated in earlier UT studies. Thus, it may be that Experiment-3 participants were less motivated than the ones included in the earlier experiments.

However, furthermore challenging the UTT, we found that unconscious thought participants did not produce significantly better apartment evaluations compared to immediate-evaluation participants in Experiment 2. This might either be an issue of statistical power as participants in the unconscious-thought condition with undemanding-distraction showed numerically better apartment-task performance than immediate-evaluation participants. Or, as for example Waroquier et al. (2010) stated, it might be just as good to trust your first intuition as to think unconsciously. However, studies with larger sample sizes are needed to resolve this issue. It further needs to be determined whether immediate-evaluation participants actually simply “trust their gut feeling” or rely on analytical thoughts or heuristics to make their evaluations.

### Thought Processes Within a UTT Paradigm

Current mind-wandering research has been applying self-report methods such as online thought probes or retrospective questionnaires. These instruments allow scientists to assess participants’ internal thought processes, which is the reason why we implemented them within a standard UTT paradigm. We believed that an insight into participants’ thoughts during unconscious- as well as conscious-thought periods might help us to better understand the processes leading to decisions within a complex-problem scenario.

Across all three experiments, we found that conscious-thought instructions indeed led to considerably higher levels of mental occupation with a previously presented evaluation problem. Still, participants did not use all of the available time for conscious problem deliberation. Indeed, they spent roughly about half of the time thinking about the problem and the other half thinking about unrelated matters. It appeared as if the deliberation time had been too long so that people’s minds started wandering. However, Experiment 2 ruled out the possibility that participants in a conscious condition would overthink the problem at hand, possibly deteriorating evaluation quality: Participants in the conscious-thought condition performed similarly well as participant in the immediate-evaluation condition. However, this also implies that high amounts of conscious thought about the evaluation problem did not lead to better evaluations compared to those formed only by first impressions, so that one could go so far as to describe conscious thought as unnecessary. In addition, the results of Experiments 1 and 2 revealed that conscious-thought participants showed worse performances evaluating the apartments than unconscious-thought participants (unless thought probed), who showed significantly lower levels of problem-related thought. Only taking into account these two experiments, one could indeed argue that lower levels of problem deliberation foster higher quality evaluations, as is assumed by the UTT. However, the results from Experiment 3 put this notion into perspective, as evaluation performance did not differ between groups, although the extent of problem deliberation differed.

In a survey concerning real life purchase decisions, Dijksterhuis et al. (2006) asked participants how much they had thought about a product they had recently bought. For complex products, the authors found that a higher amount of conscious product-thought was associated with lower satisfaction with the product. To directly test for such an association within our paradigm, we correlated the amount of problem-related thought with problem-solution quality within the experimental conditions in Experiment 3. These correlations were around zero (and not significant). That is, we did not find evidence for a relationship between the amount of ATs and decision performance within conditions on an individual level. It may still be, however, that the individual variations in ATs within each condition were just too small to affect decision performance.

In the present work, we constantly found that distraction-tasks with high demands did not leave as much room for mind wandering, problem-related or unrelated, as tasks with low demands. Such effects of task demands are found to be stable within the mind-wandering literature (e.g., Rummel and Boywitt, 2014) and suggest that task demands compete with wandering thoughts for attentional resources. Concerning a UTT paradigm, our results suggest that demanding filler-interval tasks occupy attentional resources to a higher degree than undemanding tasks, leading to fewer conscious problem-related thoughts. Before, we considered wandering thoughts as an alternative explanation for UTEs and argued that high filler-task demands would compete with adaptive mind-wandering processes for attentional resources. Further, we argued that without engagement in productive mind wandering toward the pending evaluation problem, the benefit of a distraction-task period would be reduced. Although the results within the different unconscious-thought conditions mirrored the first part of this line of reasoning, there was no evidence for a connection between higher amounts of problem-related mind wandering and better evaluations, qualifying our alternative explanation for UTEs. Yet, because working on any kind of distraction resulted in lower amounts of problem-related thoughts as well as better evaluations than engaging in conscious problem thought (at least in Experiments 1 and 2) in general, the possibility remains that “less is better,” or that there is “just the right amount” of problem-related thought which is necessary and sufficient for good decision making. Research showing that self-paced conscious thought periods are shorter than experimenter-paced conscious thought periods whilst also leading to better decisions supports this assumption (Payne et al., 2008).

Furthermore, the question remains whether what we called ATs in all our experimental conditions is qualitatively the same across conditions. Instructing participants to consciously think about a solution to the apartment problem might lead to other kinds of thought than asking them to work on a letter-task. Conscious-thought participants might have actively tried to engage in various strategies such as remembering attributes and weighting them, which might not be the best strategy within a complex decision situation given the conscious’ system’s low information-processing capacity. By contrast, while working on a distraction-task, ATs might have been of a completely different nature, possibly more focused on a holistic visualization of or a feeling evoked by a respective apartment. Such potential differences of qualitative nature should be addressed in further research, which could possibly employ qualitative methods and more detailed thought reports.

Finally, as there was no stable relation between thought condition and performance on the apartment task across all three experiments, no strong conclusions should be drawn concerning the engagement of mind-wandering processes regarding problem-deliberation and their success for complex-problem solving. With our experiments being the first using mind-wandering measures within UTE designs, further research is needed to look into the relation between different thought modes and the UTE. This includes the variation of the experimental paradigm, for example by including a mere distraction condition without the intention to further process the apartments (Bos et al., 2008), which should influence mind-wandering levels as well as the UTE^8^.

### Effects of Thought Awareness

Another issue which we addressed in the present work was whether UTEs are really the result of deliberation without attention (Dijksterhuis et al., 2006). Strick et al. (2011) already suggested replacing this term with the term deliberation-without-consciousness, focusing on the distinction between attention and consciousness, or rather awareness. Participants of an unconscious-thought experiment might allocate attentional resources toward the active problem during a filler interval, without being aware of this process. The same is true for mind wandering in general, which is found to occur with and without awareness (Smallwood and Schooler, 2006). By applying thought probes in Experiment 2, we aimed to capture both aware as well as unaware thoughts in order to relate them to problem-evaluation quality. The results indicated that self-reports from online thought probes mirrored those from retrospective questionnaires, thereby validating each other. After and during task completion, participants seem to be well aware of their recent thought processes, when asked. Although both mind-wandering assessment methods produced similar estimates on thought variables, evaluation performance varied between the two conditions which differed in nothing but the mind-wandering assessment method. A distinction between the two kinds of assessment might be that thought probes are intrusive and might bring thought processes into awareness during the task. A lack of thought awareness might, however, be a necessary criterion for an UTE to appear, which would explain the increase in evaluation performance for the condition without thought probes only. However, results from Experiment 3, which was supposed to test this assumption, were inconclusive.

Depending on the choice of mind-wandering assessment method, results of studies applying thought probes have been found to fluctuate (Seli et al., 2013; Weinstein, 2018; Weinstein et al., 2018). Given that changing probe characteristics can lead to considerable differences in results, one might argue that the mere presence of probes produces similar or even larger discrepancies. As we recently found in our lab (Steindorf et al., 2020), probing participants’ thoughts during an incubation interval within a creativity task resulted in fewer creativity-task-related thoughts (reported retrospectively) compared to not applying any probes or applying trivia probes (cf. present Experiment 3). Because trivia probes interrupt participants just as thought probes do, this effect cannot be attributed to mere task-interruption. We interpreted the findings as an awareness-effect: Thought probes might have made participants more aware and more cautious of their mind-wandering behavior, thereby changing the experience itself. Also, in the present work’s third experiment, thought reports were affected by differences in mind-wandering assessment methods. Participants who were thought-probed retrospectively reported more general TUTs compared to those who were trivia-probed or not probed at all. Asking people about their current thoughts might either change their in-the-moment mind wandering behavior or their recollection of mind-wandering instances when asked after task completion. Further research will be needed to investigate the potentially intrusive nature of thought probes for complex decisions in more detail.

To gain further insights into thought-awareness processes during UTT experiments, further research could rely on self-caught mind-wandering assessment. Employing such methods, a researcher asks participants to monitor their awareness of mind-wandering instances (Cunningham et al., 2000). Unlike probe-caught mind wandering, it requires participants to be aware of their thought experiences (Smallwood and Schooler, 2006). Employing both measures within one and the same experiment could help disentangle actual mind-wandering instances and awareness of such instances (e.g., Sayette et al., 2009).

### Conclusion

Across three experiments, we examined the nature of thoughts during UTT experiments. We relied on retrospective mind-wandering questionnaires as well as on online thought probes (i.e., methods used in current mind-wandering research) to gain insights into participants’ cognitive processes during distraction and conscious-thought periods. We demonstrated that typical UTT manipulations within the here applied apartment task change the participants’ though contents. When they were instructed to consciously think about the apartments, they spent a lot of time doing so. Distracting them from thinking about the apartments – especially with difficult filler tasks – led to considerably smaller amounts of apartment thoughts. These results represent successful critical tests of the UTT’s fundamental assumptions. Thought reports further demonstrated that filler intervals in the conscious-thought conditions do not seem to represent episodes of uninterruptedly ongoing conscious apartment-thought. Similarly, filler intervals in the unconscious-thought conditions do not seem to represent episodes of pure unconscious thought. Participants seem to experience a mixture of different thought modes, which does not rule out the possibility of unconscious thought being the driving force behind the UTE. Although we theoretically argued for mind-wandering processes as an alternative explanation for UTEs, our results do not support this notion. Both ATs and TUTs did not relate to the performance on the apartment task across all experiments as well as in the joint analyses.

Finally, we found (some) evidence for the existence of an UTE in the first two experiments. Results of the third experiments were inconclusive. As the debate concerning the existence and nature of UTEs is still ongoing, our experiments represent important pieces for the puzzle that is the UTT.

## Data Availability Statement

The datasets presented in this study can be found in online repositories. The names of the repository/repositories and accession number(s) can be found below: our data are available under https://osf.io/4375q/ (doi: 10.17605/OSF.IO/4375Q).

## Ethics Statement

Ethical review and approval was not required for the study on human participants in accordance with the local legislation and institutional requirements. The patients/participants provided their written informed consent to participate in this study.

## Author Contributions

All authors developed the initial research idea and the experiments reported here. LS ran the statistical analysis and drafted the manuscript. JR and CDB edited and commented on the draft.

## Conflict of Interest

The authors declare that the research was conducted in the absence of any commercial or financial relationships that could be construed as a potential conflict of interest.

## Footnotes

1 We thank Arndt Bröder and his lab at the University of Mannheim, Germany for sharing their materials as well as their expertise concerning the implementation of the apartment task with us.

2 We analyzed the absolute amount of ATs and TUTs to test if they would vary across groups and be related to the performance in the apartment task. An alternative variable of interest, however, might be the relative propensity with which people engaged in apartment thoughts when they were mind wandering. Therefore, we also conducted analyses of relative AT proportions, which can be found in the Supplementary Material under https://osf.io/4375q/ (doi: 10.17605/OSF.IO/4375Q).

3 Because university affiliation did not affect our measures of interest, we omitted this factor from all analyses for the sake of clarity.

4 Calculating percentages from the total values yielded 10.50% TUTs and 4.50% ATs out of 100% for participants in the condition with demanding distraction including thought probes. In the condition with undemanding distraction, 29.25% TUTs and 11.50% ATs were reported.

5 Because university affiliation did not affect our measures of interest (except for a main effect on the distraction-task performance), we collapsed across this factor for all further analyses.

6 While running the experiment, we added a fifth condition and collected data from 42 additional participants in this condition with *modified thought probes*. Only slightly deviating from the procedure with regular thought probes, participants in the condition with modified thought probes were supposed to categorize their current thoughts’ content as being task-related (related to the 1-back task), related to their task performance, or task-unrelated. That is, we did not mention the apartment task in any of the response options to the modified thought probes. This group’s data [apartment-task performance (*M* = 3.00, *SD* = 0.61), TUTs (*M* = 18.64, *SD* = 16.54), ATs (*M* = 9.83, *SD* = 11.84)] will not be further considered, because it did not deviate from the regular online thought-probe condition and we thus would not have gained any new insights from adding it to our design.

7 The relative proportion of ATs to overall mind wandering (reflecting the propensity with which people engaged in apartment thoughts when mind wandering) was significantly lower for participants receiving thought probes than for all other unconscious-thought conditions (see Supplementary Material under https://osf.io/4375q/).

8 We thank one of our reviewers for pointing this out.
