# Supplementary material for: A Fresh Look at the Unconscious Thought Effect: Using Mind-Wandering Measures to Investigate Thought Processes in Decision Problems With High Information Load
Source: Front Psychol. 2021 Jun 24;12:545928. doi: 10.3389/fpsyg.2021.545928 (PMC8264051; doi:10.3389/fpsyg.2021.545928)
Supplement: Supplementary file 1 [file Table_1.DOCX]

**Supplementary Analyses**

A fresh look at the Unconscious Thought Effect: Using mind-wandering measures to investigate thought processes in decision problems with high information load

Lena Steindorf^1^, Jan Rummel^1^, & C. Dennis Boywitt^2^

^1^Heidelberg University

^2^without affiliation

**Supplement**

*Full correlation tables for each experimental condition from Experiments 1 to 3 as well as the joint data set.*

**Experiment 1**

**Table 1**

*Pearson correlations between all measures of interest in the* ***conscious-thought condition*** *(n = 48) of Experiment 1.*

| Measure | 1 | 2 | 3 |
| --- | --- | --- | --- |
| 1. Apartment-Task Performance | - | -.05 | .04 |
| 2. TUTs (Retrospective) |  | - | - |
| 3. ATs (Retrospective) |  |  | - |

*Note.* TUTs = task unrelated thoughts, ATs = apartment thoughts. In the conscious-thought condition, TUTs directly result from ATs (and vice versa, TUTs = 100 % - ATs), which is why we do not report a correlation between them. Both reported correlations were non-significant (all *p*s > .756) and their absolute values slightly deviate from each other because one participant’s thought reports added up to 90 instead of 100%.

**Table 2**

*Pearson correlations between all measures of interest in* ***the unconscious-thought condition with undemanding distraction*** *(n = 49) of Experiment 1.*

| Measure | 1 | 2 | 3 | 4 |
| --- | --- | --- | --- | --- |
| 1. Apartment-Task Performance | - | .14 | .11 | .12 |
| 2. TUTs (Retrospective) |  | - | -.02 | -.25 |
| 3. ATs (Retrospective) |  |  | - | -.11 |
| 4. Distraction-Task Performance |  |  |  | - |

*Note.* TUTs = task unrelated thoughts, ATs = apartment thoughts. All reported correlations were non-significant (all *p*s > .081).

**Table 3**

*Pearson correlations between all measures of interest in the* ***unconscious-thought condition with demanding distraction*** *(n = 48) of Experiment 1.*

| Measure | 1 | 2 | 3 | 4 |
| --- | --- | --- | --- | --- |
| 1. Apartment-Task Performance | - | -.12 | .14 | -.13 |
| 2. TUTs (Retrospective) |  | - | .16 | **-.42**** |
| 3. ATs (Retrospective) |  |  | - | -.12 |
| 4. Distraction-Task Performance |  |  |  | - |

*Note.* TUTs = task unrelated thoughts, ATs = apartment thoughts. Bold values highlight significant correlations. * *p* ≤ .050, ** *p* ≤ .010.

**Experiment 2**

**Table 3**

*Pearson correlations between all measures of interest in the* ***immediate-evaluation condition*** *(n = 59) of Experiment 2.*

| Measure | 1 | 2 | 3 | 4 | 5 | 6 |
| --- | --- | --- | --- | --- | --- | --- |
| 1. Apartment-Task Performance | - | -.06 | -.13 | .18 | .01 | -.11 |
| 2. TUTs (Retrospective) |  | - | **.80**** | .11 | -.06 | .17 |
| 3. TUTs (Thought-Probe Report) |  |  | - | .34 | .15 | .03 |
| 4. ATs (Retrospective) |  |  |  | - | **.62**** | -.02 |
| 5. ATs (Though-Probe Report) |  |  |  |  | - | -.12 |
| 6. Distraction-Task Performance |  |  |  |  |  | - |

*Note.* TUTs = task unrelated thoughts, ATs = apartment thoughts. Because we had retrospective thought data available for participants of one data-collection location only (see main text), all correlations with retrospective TUTs and ATs are based on *n* = 28. Bold values highlight significant correlations. * *p* ≤ .050, ** *p* ≤ .010.

**Table 4**

*Pearson correlations between all measures of interest in* ***conscious-thought condition*** *(n = 61) of Experiment 2.*

| Measure | 1 | 2 | 3 |
| --- | --- | --- | --- |
| 1. Apartment-Task Performance | - | -.30 | .30 |
| 2. TUTs (Retrospective) |  | - | **-** |
| 3. ATs (Retrospective) |  |  | - |

*Note.* TUTs = task unrelated thoughts, ATs = apartment thoughts. Both reported correlations were not significant (both *p*s = .118) and were based on *n* = 29 because we had retrospective thought data available for participants of one data-collection location only (see main text). In the conscious-thought condition, TUTs directly result from ATs (and vice versa, TUTs = 100 % - ATs), which is why we do not report a correlation between them and the absolute values of the correlations with the apartment-task performance are identical.

**Table 5**

*Pearson correlations between all measures of interest in the* ***unconscious-thought condition with undemanding distraction without thought probes*** *(n = 63) of Experiment 2.*

| Measure | 1 | 2 | 3 | 4 |
| --- | --- | --- | --- | --- |
| 1. Apartment-Task Performance | - | -.19 | .15 | -.07 |
| 2. TUTs (retrospective) |  | - | -.25 | .25 |
| 3. ATs (retrospective) |  |  | - | **-.39*** |
| 4. Distraction-Task Performance |  |  |  | - |

*Note.* TUTs = task unrelated thoughts, ATs = apartment thoughts. Because we had retrospective thought data available for participants of one data-collection location only (see main text), all correlations with retrospective TUTs and ATs are based on *n* = 30. Bold values highlight significant correlations. * *p* ≤ .050, ** *p* ≤ .010.

**Table 6**

*Pearson correlations between all measures of interest in the* ***unconscious-thought condition with undemanding distraction with thought probes*** *(n = 60) of Experiment 2.*

| Measure | 1 | 2 | 3 | 4 | 5 | 6 |
| --- | --- | --- | --- | --- | --- | --- |
| 1. Apartment-Task Performance | - | .07 | .12 | .23 | -.12 | .07 |
| 2. TUTs (Retrospective) |  | - | **.69**** | -.18 | -.21 | -.19 |
| 3. TUTs (Thought-Probe Report) |  |  | - | -.25 | -.16 | -.17 |
| 4. ATs (Retrospective) |  |  |  | - | **.72**** | -.36 |
| 5. ATs (Though-Probe Report) |  |  |  |  | - | -.12 |
| 6. Distraction-Task Performance |  |  |  |  |  | - |

*Note.* TUTs = task unrelated thoughts, ATs = apartment thoughts. Because we had retrospective thought data available for participants of one data-collection location only (see main text), all correlations with retrospective TUTs and ATs are based on *n* = 29. Bold values highlight significant correlations. * *p* ≤ .050, ** *p* ≤ .010.

**Table 7**

*Pearson correlations between all measures of interest in the* ***unconscious-thought condition with demanding distraction with thought probes*** *(n = 60) of Experiment 2.*

| Measure | 1 | 2 | 3 | 4 | 5 | 6 |
| --- | --- | --- | --- | --- | --- | --- |
| 1. Apartment-Task Performance | - | .12 | -.06 | .10 | .01 | .05 |
| 2. TUTs (Retrospective) |  | - | **.68**** | .10 | -.22 | **-.40*** |
| 3. TUTs (Thought-Probe Report) |  |  | - | .00 | -.04 | **-.33**** |
| 4. ATs (Retrospective) |  |  |  | - | **.61**** | .31 |
| 5. ATs (Though-Probe Report) |  |  |  |  | - | -.09 |
| 6. Distraction-Task Performance |  |  |  |  |  | - |

*Note.* TUTs = task unrelated thoughts, ATs = apartment thoughts. Because we had retrospective thought data available for participants of one data-collection location only (see main text), all correlations with retrospective TUTs and ATs are based on *n* = 28. Bold values highlight significant correlations. * *p* ≤ .050, ** *p* ≤ .010.

**Experiment 3**

**Table 8**

*Pearson correlations between all measures of interest in the* ***conscious-thought condition*** *(n = 96) of Experiment 3.*

| Measure | 1 | 2 | 3 |
| --- | --- | --- | --- |
| 1. Apartment-Task Performance | - | .12 | -.12 |
| 2. TUTs (retrospective) |  | - | - |
| 3. ATs (retrospective) |  |  | - |

*Note.* TUTs = task unrelated thoughts, ATs = apartment thoughts. Both reported correlations were non-significant (both *p*s = .242). In the conscious-thought condition, TUTs directly result from ATs (and vice versa, TUTs = 100 % - ATs), which is why we do not report a correlation between them and the absolute values of the correlations with the apartment-task performance are identical.

**Table 9**

*Pearson correlations between all measures of interest in* ***unconscious-thought condition with undemanding distraction without thought probes*** *(n = 92) of Experiment 3.*

| Measure | 1 | 2 | 3 | 4 |
| --- | --- | --- | --- | --- |
| 1. Apartment-Task Performance | - | **-.24*** | -.06 | .16 |
| 2. TUTs (retrospective) |  | - | .08 | **-.40**** |
| 3. ATs (retrospective) |  |  | - | .06 |
| 4. Distraction-Task Performance |  |  |  | - |

*Note.* TUTs = task unrelated thoughts, ATs = apartment thoughts. Bold values highlight significant correlations. * *p* ≤ .050, ** *p* ≤ .010.

**Table 10**

*Pearson correlations between all measures of interest in the* ***unconscious-thought condition with undemanding distraction with thought probes*** *(n = 94) of Experiment 3.*

| Measure | 1 | 2 | 3 | 4 | 5 | 6 |
| --- | --- | --- | --- | --- | --- | --- |
| 1. Apartment-Task Performance | - | -.11 | .11 | .04 | -.07 | .02 |
| 2. TUTs (Retrospective) |  | - | **.73**** | **-.21*** | -.07 | .01 |
| 3. TUTs (Thought-Probe Report) |  |  | - | **-.27**** | -.20 | .07 |
| 4. ATs (Retrospective) |  |  |  | - | **.59**** | -.13 |
| 5. ATs (Though-Probe Report) |  |  |  |  | - | -.14 |
| 6. Distraction-Task Performance |  |  |  |  |  | - |

*Note.* TUTs = task unrelated thoughts, ATs = apartment thoughts. Bold values highlight significant correlations. * *p* ≤ .050, ** *p* ≤ .010.

**Table 11**

*Pearson correlations between all measures of interest in the* ***unconscious-thought condition with undemanding distraction with trivia probes*** *(n = 92) of Experiment 3.*

| Measure | 1 | 2 | 3 | 4 |
| --- | --- | --- | --- | --- |
| 1. Apartment-Task Performance | - | -.04 | .04 | -.11 |
| 2. TUTs (retrospective) |  | - | -.11 | .03 |
| 3. ATs (retrospective) |  |  | - | -.04 |
| 4. Distraction-Task Performance |  |  |  | - |

*Note.* TUTs = task unrelated thoughts, ATs = apartment thoughts. All reported correlations were non-significant (all *p*s > .305).

**Joint Analysis**

Having employed the same conscious thought and the same unconscious thought (undemanding distraction without thought probes) condition in all three experiments allowed us to collapse the data for these conditions to conduct joined analyses.

**Table 12**

*Pearson correlations between all measures of interest in all* ***conscious-thought conditions*** *(n = 205) of Experiments 1-3.*

| Measure | 1 | 2 | 3 |
| --- | --- | --- | --- |
| 1. Apartment-Task Performance | - | -.05 | .05 |
| 2. TUTs (retrospective) |  | - | - |
| 3. ATs (retrospective) |  |  | - |

*Note.* TUTs = task unrelated thoughts, ATs = apartment thoughts. Both reported correlations were non-significant (both *p*s > .495). In the conscious-thought condition, TUTs directly result from ATs (and vice versa, TUTs = 100 % - ATs), which is why we do not report a correlation between them and the absolute values of the correlations with the apartment-task performance are identical. Because for Experiment 2 we had retrospective thought data available for participants of one data-collection location only (see main text), all correlations with retrospective TUTs and ATs are based on *n* = 173.

**Table 13**

*Pearson correlations between all measures of interest in all* ***unconscious-thought condition with undemanding distraction without thought probes*** *(n = 203) of Experiments 1-3.*

| Measure | 1 | 2 | 3 |
| --- | --- | --- | --- |
| 1. Apartment-Task Performance | - | -.10 | .04 |
| 2. TUTs (retrospective) |  | - | -.01 |
| 3. ATs (retrospective) |  |  | - |

*Note.* TUTs = task unrelated thoughts, ATs = apartment thoughts. All reported correlations were non-significant (both *p*s > .211).. Because for Experiment 2 we had retrospective thought data available for participants of one data-collection location only (see main text), all correlations with retrospective TUTs and ATs are based on *n* = 170.
