# Supplementary material for: A Fresh Look at the Unconscious Thought Effect: Using Mind-Wandering Measures to Investigate Thought Processes in Decision Problems With High Information Load
Source: Front Psychol. 2021 Jun 24;12:545928. doi: 10.3389/fpsyg.2021.545928 (PMC8264051; doi:10.3389/fpsyg.2021.545928)
Supplement: Supplementary file 2 [file Table_2.DOCX]

**Supplementary Analyses**

A fresh look at the Unconscious Thought Effect: Using mind-wandering measures to investigate thought processes in decision problems with high information load

Lena Steindorf^1^, Jan Rummel^1^, & C. Dennis Boywitt^2^

^1^Heidelberg University

^2^without affiliation

**Analyses of the proportion of apartment thoughts to task-unrelated thoughts**

In our article, we report analyses for the absolute amount of apartment thoughts (ATs) and task-unrelated thoughts (TUTs). Another interesting variable however, is the relative proportion of ATs to overall mind wandering as it would reflect the propensity with which people engaged in apartment thoughts when mind wandering. Therefore, we report supplementary analyses for this measure (ATs/(ATs+TUTs)). *N*s might vary between the here reported results and results in the main article, because of some participants not reporting any ATs and TUTs at all, making a division impossible and thus resulting in missing values.

Table 1 depicts descriptive statistics for the proportion of ATs to TUTs for all experiments and conditions. In Experiment 1, there was a significant main effect of the experimental condition, *F*(2, 133) = 3.66, *p* = .028, with participants in the unconscious-thought condition with demanding distraction showing a smaller proportion of relative ATs than those with undemanding distraction (*p* = -.018) and those in the conscious-thought condition (*p* = -.033). There was no significant difference between the latter two conditions (*p* = .068). Thus, results reflected the participants’ task on the one hand with a high proportion of ATs when participants were supposed to think about the apartment. On the other hand, for unconscious-thought participants, the proportion was smaller when the current n-back task was more demanding.

In Experiment 2 (Heidelberg participants only), there was no significant main effect of the experimental condition, *F*(4, 134) = 2.16, *p* = .077.

In Experiment 3, there was a significant main effect of the experimental condition, *F*(3, 353) = 10.52, *p* < .001. Conscious-thought participants reported the highest proportion of ATs to TUT compared to all other conditions (all *p*s < .017). Unconscious-thought participants who had received thought probes reported the lowest proportion of relative ATs (all *p*s < .030). Thus, as in Experiment 1, results reflected the participants’ task with a high proportion of relative ATs when participants were supposed to think about the apartment. Further, receiving thought probes resulted in lower relative ATs compared to not receiving them.

To more directly test for a relation between relative ATs during the filler interval and later apartment-task performance, we correlated these measures within conditions. Relative ATs did not correlate with the apartment-task performance in any of the experimental conditions, all ps > .242.

Table 1

| Experiment/condition |  | immediate evaluation | conscious thought | demanding distraction | demanding distraction with thought probes | undemanding distraction | undemanding distraction with thought probes | undemanding distraction with trivia probes |
| --- | --- | --- | --- | --- | --- | --- | --- | --- |
| Experiment 1 | ***M*** |  | **0.47** | **0.31** |  | **0.46** |  |  |
|  | *SD* |  | 0.25 | 0.36 |  | 0.34 |  |  |
| Experiment 2 | ***M*** | **0.22** | **0.38** |  | **0.22** | **0.40** | **0.30** |  |
|  | *SD* | 0.31 | 0.26 |  | 0.29 | 0.39 | 0.29 |  |
| Experiment 3 | ***M*** |  | **0.54** |  |  | **0.43** | **0.29** | **0.39** |
|  | *SD* |  | 0.23 |  |  | 0.34 | 0.29 | 0.35 |

*Means (M) and Standard Deviations (SD) for the Proportion of ATs to TUTs for all Experiments and Conditions*
